# Automated brain segmentation and volumetry in dementia diagnostics: a narrative review with emphasis on FreeSurfer

**DOI:** 10.3389/fnagi.2024.1459652

**Published:** 2024-09-03

**Authors:** Eya Khadhraoui, Thomas Nickl-Jockschat, Hans Henkes, Daniel Behme, Sebastian Johannes Müller

**Affiliations:** ^1^Clinic for Neuroradiology, University Hospital, Magdeburg, Germany; ^2^Department of Psychiatry and Psychotherapy, University Hospital, Magdeburg, Germany; ^3^German Center for Mental Health (DZPG), Partner Site Halle-Jena-Magdeburg, Magdeburg, Germany; ^4^Center for Intervention and Research on Adaptive and Maladaptive Brain Circuits Underlying Mental Health (C-I-R-C), Magdeburg, Germany; ^5^Neuroradiologische Klinik, Katharinen-Hospital, Klinikum-Stuttgart, Stuttgart, Germany; ^6^Stimulate Research Campus Magdeburg, Magdeburg, Germany

**Keywords:** dementia, FreeSurfer, segmentation, volumetry, review

## Abstract

**Background:**

Dementia can be caused by numerous different diseases that present variable clinical courses and reveal multiple patterns of brain atrophy, making its accurate early diagnosis by conventional examinative means challenging. Although highly accurate and powerful, magnetic resonance imaging (MRI) currently plays only a supportive role in dementia diagnosis, largely due to the enormous volume and diversity of data it generates. AI-based software solutions/algorithms that can perform automated segmentation and volumetry analyses of MRI data are being increasingly used to address this issue. Numerous commercial and non-commercial software solutions for automated brain segmentation and volumetry exist, with FreeSurfer being the most frequently used.

**Objectives:**

This Review is an account of the current situation regarding the application of automated brain segmentation and volumetry to dementia diagnosis.

**Methods:**

We performed a PubMed search for “FreeSurfer AND Dementia” and obtained 493 results. Based on these search results, we conducted an in-depth source analysis to identify additional publications, software tools, and methods. Studies were analyzed for design, patient collective, and for statistical evaluation (mathematical methods, correlations).

**Results:**

In the studies identified, the main diseases and cohorts represented were Alzheimer’s disease (*n* = 276), mild cognitive impairment (*n* = 157), frontotemporal dementia (*n* = 34), Parkinson’s disease (*n* = 29), dementia with Lewy bodies (*n* = 20), and healthy controls (*n* = 356). The findings and methods of a selection of the studies identified were summarized and discussed.

**Conclusion:**

Our evaluation showed that, while a large number of studies and software solutions are available, many diseases are underrepresented in terms of their incidence. There is therefore plenty of scope for targeted research.

## 1 Introduction

According to the WHO, dementia is currently the seventh most common cause of death and one of the leading causes of disability and dependency among older people worldwide (World Health Organization, 2017). Furthermore, its incidence is likely to increase in coming years caused by aging populations. Accordingly, its early detection and prevention are matters of increasing urgency, necessitating methods for accurate diagnosis of the underlying disease. Diagnosis of dementia by clinical examination is often inconsistent and subject to inaccuracy. Additional biomarkers, such as cerebrospinal fluid (CSF) and positron emission tomography (PET), are often not groundbreaking either. However, magnetic resonance imaging (MRI) enables reliable and unambiguous classification of brain status.

Current high-resolution MRI is performed using magnetic field strengths of up to 7 Tesla, enabling excellent representations of brain tissue. However, the enormous amounts of image data generated present an obstacle to thorough analysis. An increasingly common method to address this obstacle is the use of computer software capable of automated MRI volumetry, whereby the volumes of specific anatomic brain regions are calculated using segmentation algorithms and detailed atlases.

Such segmentation tools enable a fully automated and objective assessment of brain atrophy. The results can confirm suspected diagnoses or provide differential diagnoses. Standardized use can also save time in radiological reporting.

Currently, one of the first and most recognized software solutions is FreeSurfer, (Fischl, 2012) with 2,925 results being returned on PubMed using the search string “FreeSurfer.” It performs calculations lasting hours to days to produce robust and reliable results. For comparison, its “little brother” FastSurfer (Henschel et al., 2020) only returns 16 results on PubMed (search string “FastSurfer”).

The increasing prevalence of high-resolution sequences and 7-Tesla MRI could lead to problems for software solutions based on fixed-resolution or resolution-ignorant convolutional neural networks (CNNs). One possible solution is the new FastSurferVINN (Henschel et al., 2022). A slower high-resolution stream for FreeSurfer also exists (Zaretskaya et al., 2018). In any case, we are certain to see changes in the volumetry software used due to this trend in the next few years.

The aim of this review was to assess the status of automated volumetry in 2024 and identify recommendations, gaps, and opportunities within MR brain research. The focus was on FreeSurfer software and Alzheimer’s disease.

Even if global cortical surface area, thickness, and volume are not related to cognitive scores (Li et al., 2023), volumetric analysis is a useful tool to study and observe dementias. For instance, new Alzheimer medications based on antibodies against amyloid plaque can cause serious side effects leading to Amyloid-Related Imaging Abnormalities (ARIAs) or accelerated atrophy (Pinter et al., 2022), so for patients taking such medications, regular volumetric monitoring of the brain is essential (Withington and Turner, 2022; Van Dyck et al., 2023).

### 1.1 Search terms and included studies

**Figure 1:**
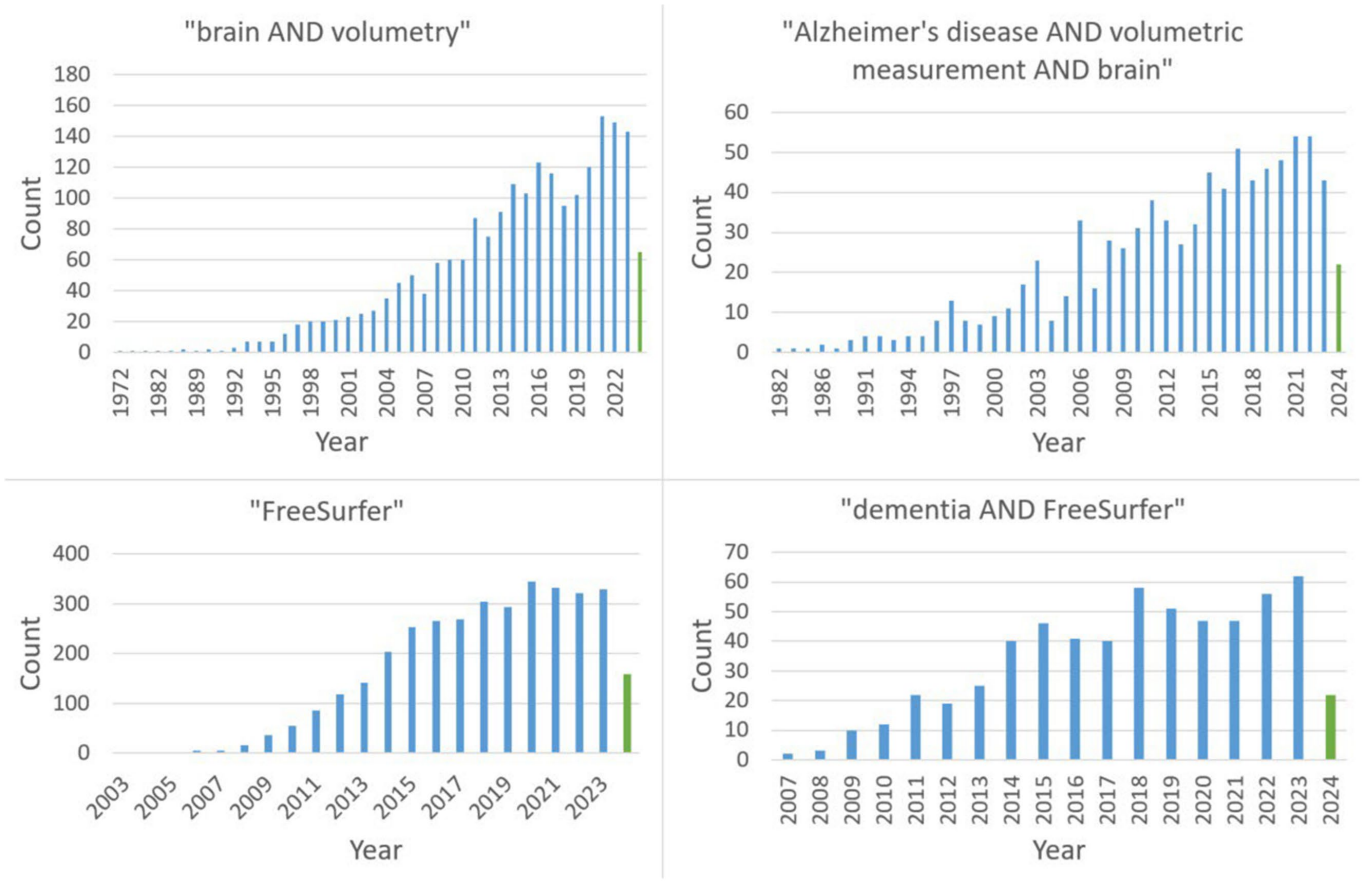
The PubMed time line of the four relevant search terms (green: 2024).

The two major search terms were “dementia AND FreeSurfer” as well as “Alzheimer’s disease AND volumetric measurements AND brain.” Figure 1 reveals the continuing trend with a steady increase (with a possible plateau formation in the last years) in publications on PubMed regarding the search queries relevant to this review.

**Figure 2:**
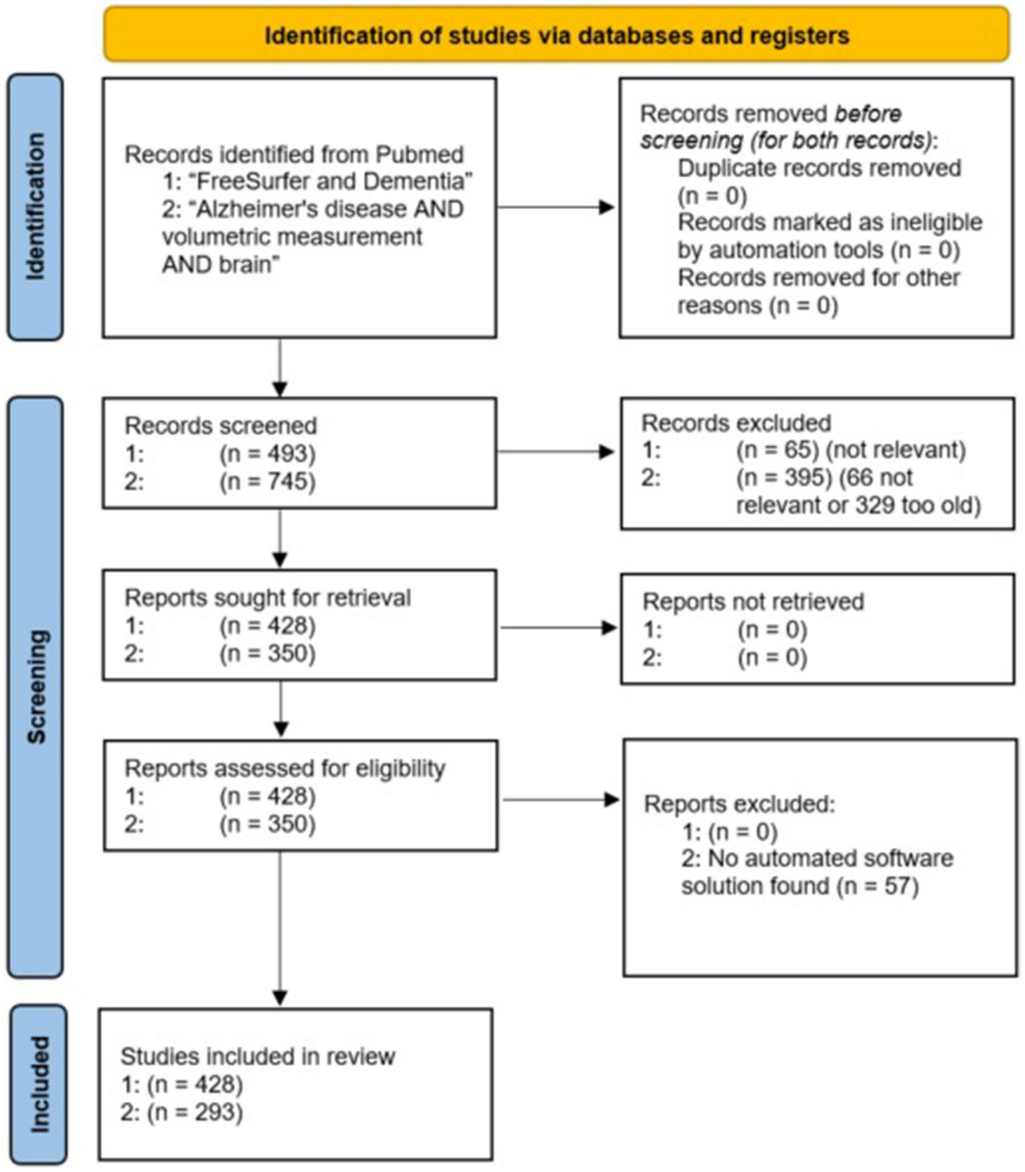
PRISMA flow chart of the included PubMed studies for both search terms.

A PRISMA flow chart (Rethlefsen and Page, 2021) of the evaluated studies is shown in Figure 2. To reduce the risk of overlooking/underestimating relevant programs, we additionally performed a deep search for all software tools found.

## 2 Current state of the art

### 2.1 Evaluated dementias

**Figure 3:**
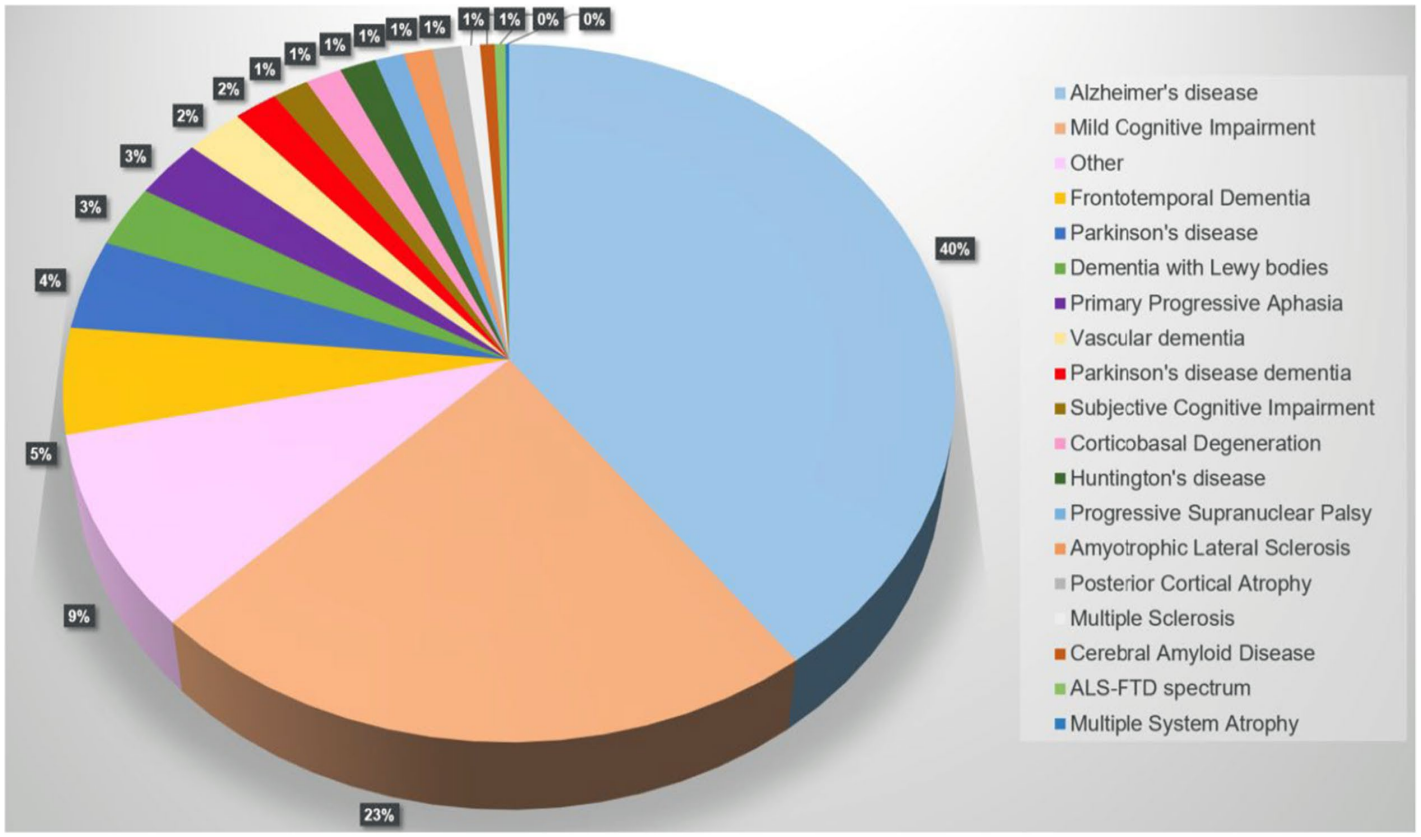
Pie chart showing the distribution of cohorts/diseases returned by the PubMed search “FreeSurfer and Dementia” (*n* = 428 of 493 studies, 01/01/2024).

A PubMed search on “FreeSurfer and Dementia” returned 493 results, 428 were included. Alzheimer’s disease (AD) was the most analyzed disease (40%), followed by mild cognitive impairment (MCI), frontotemporal dementia, and Parkinson’s disease (PD). Figure 3 illustrates the distribution of dementias evaluated using FreeSurfer.

The category “other” includes cohorts with less typical diseases or specific groups of interest in certain circumstances associated with suspected brain volume loss or fluctuations, such as HIV or Down syndrome. Supplementary Figure S1 shows the distribution of these entities. The results show that there is still a need for targeted research.

When it comes to the more common dementias, it is noticeable that the subgroupings are differently defined depending on the study. This makes a comparison, for example in the context of a meta-analysis, more difficult. More precise definitions appear to be necessary, e.g., for subgroups with mild cognitive impairment [e.g., MCI with PD or MCI before PD dementia (PDD)] or for classification into mild or severe symptoms. Table 1 shows a detailed breakdown of our PubMed search.

**Table 1:** Evaluated cohorts/diseases returned by the PubMed search “FreeSurfer and Dementia” (*n* = 428 of 493 studies, 01/01/2024).

Group	Found cohorts (*n*)	Found cohorts (%)
Sum	1,049	100.0%
Healthy / control group	356	33.9%
Alzheimer’s disease	276	26.3%
Mild cognitive impairment	157	15.0%
Other	63	6.0%
Frontotemporal dementia	34	3.2%
Parkinson’s disease	29	2.8%
Dementia with Lewy bodies	20	1.9%
Primary progressive aphasia*	19	1.8%
Vascular dementia	16	1.5%
Parkinson’s disease dementia	12	1.1%
Subjective cognitive impairment	10	1.0%
Corticobasal degeneration	10	1.0%
Huntington’s disease	10	1.0%
Progressive supranuclear palsy	8	0.8%
Amyotrophic lateral sclerosis	8	0.8%
Posterior cortical atrophy	8	0.8%
Multiple sclerosis	5	0.5%
Cerebral amyloid disease	4	0.4%
ALS-FTD spectrum	3	0.3%
Multiple system atrophy	1	0.1%

While the number of Alzheimer’s cohorts examined dominates, individual dementias are significantly underrepresented in terms of incidence; particularly dementias in which no specific atrophy pattern is expected, such as vascular dementia (VD) or dementia with Lewy bodies (DLB).

### 2.2 Volumetric software

**Figure 4:**
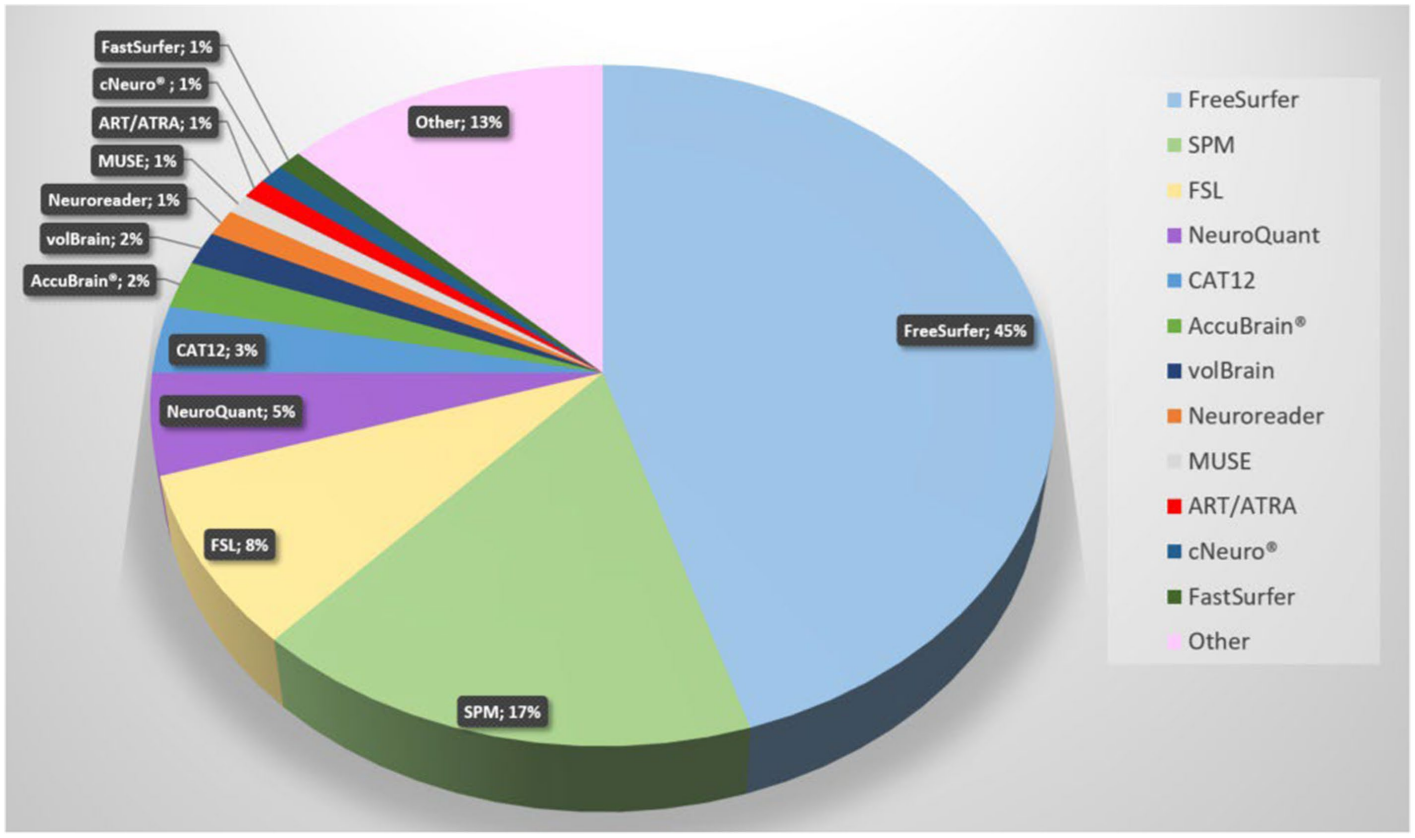
Pie chart of software solutions in reports retrieved from Pubmed with the search term “Alzheimer’s disease volumetric measurement brain” (most recent; descending). The newest 350 entries (from 2024 to 2015) were evaluated, 293 were included. All solutions with fewer than three entries are summarized under “Other”.

Since AD is the most studied dementia, we focused our search on this. A PubMed search on “Alzheimer’s disease AND volumetric measurement AND brain” revealed 745 results. The search revealed that FreeSurfer, SPM, and FSL are currently the most used software tools. Figure 4 demonstrates a pie chart of the mostly used tools. For a detailed list of software solutions see Table 2.

**Table 2:** Shows more details of a short PubMed search relating to software solutions.

Software	Developer	Task/diagnosis	%
AccuBrain®	9 Chak Cheung Street, Shatin, New Territories, Hong Kong	Whole-brain segmentation / AD, MCI, NC, FTD, diabetes Type 2, CSVD, VD, amyotrophic laterals sclerosis, HIV-associated neurocognitive disorder, hippocampal sclerosis, NMOSD	2.2%
ANALYZE software package (1989)	Mayo Foundation, Rochester, MN, USA	(obsolete) The ANALYZE7.5 file format was the basis of many new software solutions (e.g., SPM, FSL, FReeSurfer)	0.3%
ART (Automatic Registration Toolbox)	Nathan Kline Institute for Psychiatric Research, Orangeburg, NY, United States	(obsolete) NC	1.0%
ASEG	Old atlas, part of FreeSurfer	Whole-brain segmentation, hippocampal volume / epilepsy	0.3%
CAT12 (SPM12 and MathLab Lib based)	Structural Brain Mapping Group at the University of Jena, Germany	Whole-brain segmentation, hippocampal volume / epilepsy, bipolar disorder, stroke, autism, depression, PD, AD, cocaine use disorder, MS, VD, schizophrenia, NC	3.2%
CIVET	McCo nnell Brain Imaging Centre, Montreal Neurological Institute, McGill University, Montréal, Canada	Whole-brain segmentation, hippocampal volume / PD, AD, NC, drug use disorder, epilepsy, MS, autism, schizophrenia	0.3%
cNeuro®	Combinostics, Hatanpään valtatie 24 33,100 Tampere, Finland	Whole-brain segmentation, MS	1.0%
FastSurfer	Martinos Center for Biomedical Imaging and Harvard Medical School, Boston, USA	Whole-brain segmentation, MS, stroke, multiple system atrophy, DLB, PD, progressive supranuclear palsy	1.0%
FreeSurfer (no version info)		Whole-brain segmentation, hippocampal, thalamic and brain stem subsegmentations / dementia, psychiatry, MS, PD, NC, epilepsy…	15.4%
Freesurfer 4.1			1.0%
Freesurfer 4.3			1.0%
Freesurfer 5.0			1.0%
Freesurfer 5.1			6.4%
Freesurfer 5.2			0.3%
Freesurfer 5.3			8.3%
Freesurfer 6.0			9.9%
Freesurfer 7.1.1			1.9%
FSL (without further specification)	Analysis Group, FMRIB, Oxford, UK	Whole-brain segmentation, hippocampal volume, thalamic volumetry / MS, epilepsy, NC, radiotherapy-associated brain changes, psychiatric disorders, …	5.4%
FSL FAST			0.3%
FSL FIRST			2.6%
Gif geodesic information flow	M. J. Cardoso, Translational Imaging Group, Centre for Medical Image Computing (CMIC), University College London, UK	Whole-brain segmentation, basal fore brain, thalamic volumes / FTD, MS	0.6%
HAMMER	Radiology and BRIC, University of North Carolina at Chapel Hill, North Carolina, USA	Whole-brain segmentation, hippocampal volume, AD, epilepsy	0.6%
Hippodeep	Benjamin Thyreau, Tohoku University, Japan; Institute of Development, Aging and Cancer, Tohoku University, Japan	hippocampal volume / AD, epilepsy	0.3%
HIPS	Hippocampal Pipeline of volBrain	hippocampal volume / AD, epilepsy, childhood maltreatment	0.3%
icobrain dm	Icometrix, Kolonel Begaultlaan 1b / 12, 3,012 Leuven, Belgium	Whole-brain segmentation, hippocampal volume / AD, MCI, FTD epilepsy, depression	0.3%
Inbrain	MIDAS IT, Seongnam, South Korea	Whole-brain segmentation, hippocampal volume / AD, MCI, NC	0.6%
MriCloud	Center for Imaging Science (CIS), Whiting School of Engineering, Johns Hopkins University, Baltimore, USA	Whole-brain segmentation, corpus callosum, hypothalamis structures / hydrocephalus, schizophrenia, depression, AD, MCI, NC, PD	0.3%
MUSE	Center for Biomedical Image Computing and Analytics (CBICA), University of Pennsylvania, Philadelphia, USA.	Whole-brain segmentation / AD, NC	1.0%
Neuromorphometrics	Neuromorphometrics, Inc., 3 Seal Harbor Rd. PH 31, Winthrop, MA 02152–1,083 USA	Whole-brain segmentation, hippocampal volume / AD, MCI, NC, epilepsy,PD, amyotrophic laterals sclerosis, head and neck cancer survivors	0.3%
NeuroQuant	CorTechs Labs Inc. San Diego, USA	Whole-brain segmentation, hippocampal volume / AD, MCI, NC, epilepsy, schizophrenia, hypoxia, CVOD19-associated brain volume loss, MS, radiotherapy-associated brain volume loss, …	4.8%
Neuroreader	NR; Brainreader Aps, Horsens, Denmark	Whole-brain segmentation, hippocampal volume / AD, MCI, NC, FTD, epilepsy, primary progressive aphasia	1.3%
PMOD / pNEURO	Bruker’s Preclinical Imaging Division, Industriestrasse 26, 8,117 Fällanden, Switzerland	PET, SPECT and MRI, whole-brain segmentation, olfactory cortex and hippocampus / stroke,	0.6%
Quantib® ND	DeepHealth, 212 Elm St., Somerville, MA, USA	Whole-brain segmentation, white-matter lesions / FTD, AD, MCI, NC, patent foramen ovale, stroke, VD	0.6%
SAMSEG	Koen Van Leemput, available in FreeSurfer 7.2	Whole brain segmentation, contrast adaptive, white matter lesions / MS, VD	0.6%
SLANT (Spatially Localized Atlas Network Tiles)	Vanderbilt University, Nashville, TN, USA	Whole brain segmentation, 7 T / NC	0.6%
SPM	Functional Imaging Laboratory, UCL Queen Square Institute of Neurology, London, UK	Whole-brain segmentation, hippocampal volumes / dementia, psychiatry, MS, PD, NC, epilepsy…	3.8%
SPM2			0.3%
SPM3.0.4			0.3%
SPM8			3.5%
SPM12			9.0%
volBrain	ITACA, Valencia, Spain and Pictura Research Group, Bordeaux, France	Whole-brain segmentation, white matter lesions / MS, Lupus erythematous, migraine, AD, MCI, NC, …	1.6%

Of course, this overview can never be complete in such a rapidly developing environment. AD, Alzheimer’s disease; MCI, mild cognitive impairment; VD, vascular dementia; CSVD, cerebral small vessel disease; FTD, frontotemporal dementia; NC, normal control; group; DLB, dementia with Lewy bodies; MS, multiple sclerosis; NMOSD, neuromyelitis optical spectrum disorders; PD, Parkinson’s disease.

*This list does not claim to be complete, e.g., DeepBrain (VUNO Inc., Seoul, South Korea), SynthSeg + (Centre for Medical Image Computing, University College London, London, UK) or Siemens Morphometry (AI-Rad Companion; Siemens Healthineers), BrainSuite (University of California, Los Angeles and University of Southern California, California, United States) were not found by the search term.

### 2.3 Performance assessment of the segmentation software

Several statistical parameters for the measurement of accuracy and quality of segmentation tools exist. Beside sensitivity, frequently used metrics are the dice similarity coefficient (Dice, 1945; Shamir et al., 2018) (0–1, higher better). the (Pompeiu-) Hausdorff distance (HD) (Birsan and Tiba, 2006) (in mm, lower better), and the mean average precision metric (mAP; 0–1, higher better) (Beitzel et al., 2009). A further development is the Modified or Robust Hausdorff Distance (MHD, HD95) (Huttenlocher et al., 1993), which is not sensitive to local outliers.

Often, manual segmentation (or another validated gold standard) is not used as a comparison segmentation to determine these values, but another automatic segmentation (e.g., FreeSurfer).

It is not yet clear whether these statistical values, which have been adopted from other areas for the segmentation algorithms, really allow a sufficient assessment, especially in brain tumor segmentation (Hoebel et al., 2024). Especially with FreeSurfer, it is difficult to find exact current parameteres due to the rapid development, and the metrics given are often limited to certain areas of the brain and types of MRI, e.g., hippocampal volume and 7-Tesla MRI (Hosseini et al., 2016; Schmidt et al., 2018; Li and Martinez, 2020). A comparison of white matter segmentations of FreeSurfer 6, FSL 5 and SPM 12, and revealed in simulated MRI following noise level dependant result: FreeSurfer (Dice index 0.88–0.90; HD 14–35 mm; MHD 4–6 mm); FSL (Dice index 0.89–0.96; HD 20–60 mm; MHD 3–22 mm); SPM (Dice index 0.87–0.94; HD 20–25 mm; MHD 4–9 mm) (Li and Martinez, 2020).

## 3 Tailor-made software solutions for the right question

### 3.1 The (“symmetric”) healthy or aged brain

Multiple software solutions have been developed for the segmentation and volumetry of the healthy or aged brain, e.g., FreeSurfer (Fischl, 2012), FastSurfer (Henschel et al., 2020), SAMSEG (as part of FreeSurfer) (Puonti et al., 2016; Cerri et al., 2023), NeuroQuant (Ross et al., 2012; Yim et al., 2021), SynthSeg (Billot et al., 2023), DeepBrain (Suh et al., 2020), volBrain (Manjón and Coupé, 2016), inBrain (Lee J. et al., 2021; Lee J. Y. et al., 2021), CAT-12 (Gaser et al., 2022), icobrain dm (Struyfs et al., 2020), FSL (Smith et al., 2004; Woolrich et al., 2009; Jenkinson et al., 2012) (with several segmentation tools), and Siemens Morphometry (Rahmani et al., 2023).

### 3.2 The “non-healthy” brain

Algorithms for the segmentation of the asymmetrical, unhealthy brain (tumor, stroke, traumatic brain injury) are not part of this review, but should be mentioned for completeness. In these cases, sometimes a more complex segmentation is needed, because symmetric approaches and atlases as described in the section above could fail.

One of the best known representatives is DeepMedic (Kamnitsas et al., 2017). Several hundred other approaches for the segmentation of brain tumors exist, many of which are compared annually in the BRATS challenge (Kazerooni et al., 2023), although validation and evaluation have also proven to be complicated. Recently, several new approaches based on generative adversarial networks (GANs) or U-Nets have been published, e.g., MMGan (Gao et al., 2023), nnUNetFormer (Guo et al., 2023), and multi-scale context UNet-like network (Qian et al., 2024).

Brain volume loss can also be detected in several diseases in younger patients, e.g., corpus callosum and thalamus volumes can decrease in patients with multiple sclerosis (Fujimori and Nakashima, 2024). However, for these studies it must always be noted that the accuracy of some segmentation algorithms may be reduced by the presence of multiple lesions (De Sitter et al., 2020).

## 4 Anatomic regions of interest

### 4.1 Cortex and white matter

The segmentation and volumetrization of cortex and white matter is the basis of all brain volume diagnostics. Brain volume loss occurs in both aging and dementia, but it is locally or globally accelerated in most central nervous system diseases, e.g., AD (Chwa et al., 2023) or PD (Jahanshahi A. et al., 2023). Therefore, most studies require a suitable control group of the same age.

In addition, it must be mentioned that brain volume also seems to depend on diet (Karstens et al., 2019; Bramen et al., 2023). For example, body mass index and hypothalamic volume are associated, and gray-matter-volume loss is described in anorexia nervosa, (Lyall et al., 2024) while minor physiological factors, like dehydration, blood pressure, caffeine levels, and circadian rhythm, do not seem to have any influence (Zahid et al., 2022).

However, there are slight differences between individual T1 sequences and MRI scanners, leading to slight shifts between gray and white matter volume. Therefore, this phenomenon can occur when analyzing the basal ganglia.

Individual software solutions also show differences from one another in (volume) calculations; for example, the voxel-based morphometry (VBM) results by SPM and FSL and the grey matter volume results by FSL, FreeSurfer, and SPM show dissimilarities (Rajagopalan and Pioro, 2015). In an ideal study, all patients would be scanned on the same scanner with the same sequence and should be analyzed with the same reliable software tool.

### 4.2 Thalamic nuclei

An additional FreeSurfer script contains a specific atlas and enables the fine segmentation of the thalamic nuclei (Iglesias et al., 2018). These scripts also work on FastSurfer segmentations, which use the same data structures. Many other approaches also exist (Su et al., 2019; Forno et al., 2023; Pfefferbaum et al., 2023; Vidal et al., 2024).

### 4.3 Brainstem and cerebellum

There is also an additional FreeSurfer script for this specific segmentation (Iglesias et al., 2015b), but it only offers a rough subdivision. For some diseases, such as progressive supranuclear palsy (PSP), multiple system atrophy (MSA), and corticobasal syndrome, analysis of the brainstem is crucial (Brinia et al., 2023), but it is also atrophied in other dementias (Müller et al., 2023). Deep learning approaches are become more widely adopted here (Nigro et al., 2024). Some software solution can additionally analyze cerebellar hemispheres, e.g., volBrain. CerebNet (Faber et al., 2022) is compatible with FreeSurfer and FastSurfer and is able to measure cerebellar lobes.

### 4.4 Hippocampus

Since the hippocampus plays a crucial role in both AD and epilepsy, there are many approaches to its segmentation and volumetry in both dementia research and epilepsy research. Hippocampal volume can be used as early marker of dementia (Gentreau et al., 2023).

FreeSurfer provides a specific script (Iglesias et al., 2015a) for the segmentation of hippocampal subfields and the nuclei of the amygdala that supports T1-weighted and T2-weighted sequences.

**Figure 5:**
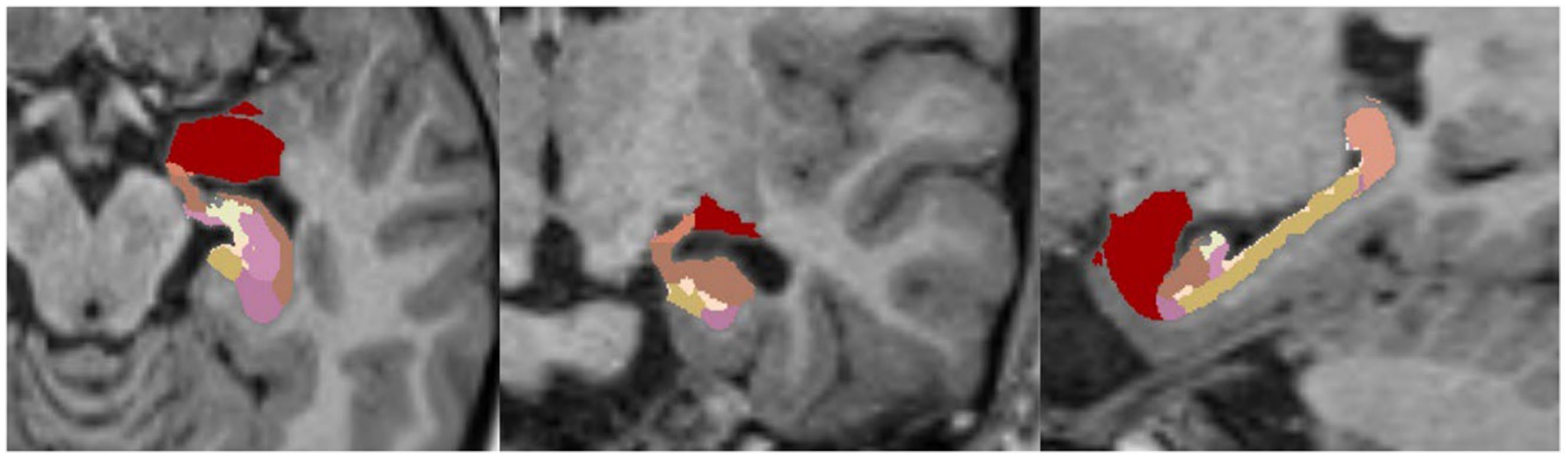
Example of FreeSurfer’s segmentation (HBT, Head Body Tail) of hippocampal subfields without nuclei of the amygdala in a 1.5-Tesla T1-MPRAGE sequence, transversal (left), coronary (middle) and sagittal (right).

An example of such a segmentation of a healthy brain/hippocampus is shown in Figure 5.

Another popular approach is the automatic segmentation of hippocampal subfields (ASHS; https://www.nitrc.org/projects/ashs), which uses three-dimensional CNNs (Goubran et al., 2020). Several studies have compared the different algorithms in patients with AD (Mueller et al., 2018; Xie et al., 2018), or across lifespans (Bender et al., 2018).

### 4.5 Cerebral networks and connectomes

Atlas-based segmentations enable the design of connection models of the human brain. Such models can be designed using graph theory approaches, and several tools have been built, e.g., Brain Connectivity Toolbox (Rubinov and Sporns, 2010), eConnectome (He et al., 2011), BRAPH (Mijalkov et al., 2017), GRETNA (Wang et al., 2015), GAT (Hosseini et al., 2012), and GraphVar (Kruschwitz et al., 2015).

In the future, comparing the connectivity models of patients with dementia with those of healthy controls could reveal new disease concepts and causes of impairments.

## 5 Mild cognitive impairment (MCI)

MCI is defined as an intermediate state (or prodromal stage) between normal aging and dementia (Petersen et al., 1999) with a wide range of heterogeneous underlying pathophysiologies. Its prevalence in older (>80 years) patients is high (Campos et al., 2024). MCI does not necessarily convert into dementia. Subjects can recover from it. Diagnosis can be established using several tests, e.g., the Montreal cognitive assessment score (Nasreddine et al., 2005; Malek-Ahmadi and Nikkhahmanesh, 2024) or minimum mental state examination (Zaudig, 1992). Another category is subjective cognitive impairment (SCI), which describes a cognitive worsening that cannot be verified by standard tests (Garcia-Ptacek et al., 2014). Such patients are usually more educated and thus likely to pass the tests because they have a higher baseline cognitive level.

MCI (or SCI in some studies) is often examined as a comparison population. In many studies, it is not entirely clear which dementia the corresponding MCI will later develop into. In some studies, MCI is also divided into subgroups (MCI-AD, MCI-FTD, etc.) depending on the study design and protocol.

Aging, MCI, and AD are related with widespread cortical and subcortical atrophy and have overlapping atrophy patterns (Chwa et al., 2023). Therefore, brain changes in MCI are subtle and show as moderate atrophies of the hippocampus and amygdala (Qu et al., 2023) as well as hypometabolism (Bailly et al., 2015). A recent study found altered cortical and subcortical morphometry and asymmetries in SCI and MCI (Yang et al., 2023). A meta-analysis revealed that differentiation of MCI and AD using the whole hippocampus volume was not significantly worse than a hippocampal subfield analysis (Zhang J. et al., 2023), mainly because atrophy patterns are not restricted to specific subfields. Therefore, a precise differentiation should be made earlier, i.e., at the SCI stage.

Asymmetry of hippocampal subfields is often present in MCI and AD (Jahanshahi A. R. et al., 2023), but its diagnostic value is still a matter of debate (Singh et al., 2023).

A large Finnish study tried to prevent cognitive impairment with a 2-year multimodal intervention (diet, exercise, cognitive training, and vascular risk monitoring). They could not find significant differences between the intervention and control groups for regional brain volume changes (Stephen et al., 2019).

## 6 Dementias

### 6.1 Alzheimer’s disease

As revealed by our PubMed search “FreeSurfer and Dementia,” AD (Stoddart, 1913) is the most common (Stoeck et al., 2012) and best-researched dementia in terms of volumetric analysis. It represents an enormous global burden (Gauthier et al., 2022). The existence of several subtypes makes precise detection by MRI methods in some cases difficult or impossible, especially in patients with hippocampal sparing patterns or without atrophy (Ferreira et al., 2017). Another limiting factor is its potential co-existence with other diseases in older patients, e.g., vascular risk factors and carotid atherosclerosis are also associated with cortical volume loss (Cardenas et al., 2012), which supports the “double hit” theory for AD.

Even though the importance of volumetry is increasing, conventional visual radiologically ratings remain a valid and reliable alternative, e.g., the medial temporal lobe atrophy scale (Molinder et al., 2021) or the entorhinal cortex atrophy (ERICA) score (Enkirch et al., 2018).

In a study by Hari et al. (2023), morphometric analysis of medial temporal lobe subregions revealed a volume reduction of the entorhinal cortex as well as of the anterior amygdaloid area in the early stages of SCI-AD and MCI-AD, which partially correlates with the pathological findings of Braak, who found the origin of neurofibrillary (Tau) pathologies in the transentorhinal and entorhinal region (as well as the hippocampus) (Braak et al., 2006). So the medial temporal lobe remains the main target for early diagnoses, even if age-related and amyloid-beta-independent tau deposition is also observed in the frontal and parietal cortical regions (Wuestefeld et al., 2023).

A study in China emphasized the importance of the volume of the presubiculum in hippocampal subfield analysis and demonstrated that a specific volume loss is associated with memory decline during the early phase and progression of AD (Xiao et al., 2023).

FreeSurfer’s Bayesian longitudinal segmentation of hippocampal substructures (Iglesias et al., 2016) performed well in two large collectives for Alzheimer’s Disease Neuroimaging Initiative (ADNI) (Mueller et al., 2005) and Minimal Interval Resonance Imaging in Alzheimer’s Disease (MIRIAD) (Malone et al., 2013). The sensitivity to distinguish between controls and patients with AD was increased. New atrophy patterns or differences in atrophy rates, e.g., in the right parasubiculum, left and right presubiculum, as wells as right subiculum, were found.

In some studies, commercial software tools, e.g., IcoBrain DM, performed partially better than FreeSurfer regarding volumetric errors, test–retest reliability, and diagnostic performance for AD (Wittens et al., 2021).

A standardized medial temporal atrophy volume ratio, which was calculated by QBraVo based on SPM8, revealed a good diagnostic performance for differentiation of AD and control group, as well as MCI and a control group (Ryu et al., 2022).

Resting-state functional connectivity and hippocampal radiomic features can also provide information about compensatory mechanisms and cognitive decline in the event of progressive volume loss of the hippocampus (Du et al., 2023).

Of course, Alzheimer’s disease spreads to many other areas of the brain over time, for example cortical thinning in the dorsal lateral prefrontal cortex and/or superior parietal cortex can be associated with a decline in cognitive-motor automaticity and task prioritization (Longhurst et al., 2023).

The complex division into multiple subclasses can be simplified using artificial intelligence methods. A study from Columbia demonstrated a possible classification of Alzheimer’s disease stages using deep learning (Mora-Rubio et al., 2023).

A method to handle the heterogeneity of AD atrophy patterns is normative modeling, with one study presenting a possible solution using multimodal variational autoencoders to identify such deviations (Kumar et al., 2023).

Another mathematical approach is the so-called graph theory, which defines the brain as a network of nodes and edges (connections), with pathologies corresponding to defects within this architecture. While the nodes usually represent specific segmented brain areas and their volumes, definition of their edges can vary from study to study, but often consists of correlations between brain areas. A study from Japan revealed left dominant morphometric changes of these networks in patients with AD (Maruoka et al., 2023). This method also enables the prognosis of epilepsy in patients with AD, as demonstrated in a study from Korea (Lee et al., 2023).

When considering Alzheimer’s disease, one should not forget that an inflammatory component is also suspected (Newcombe et al., 2018). Interestingly, a study from Japan found a negative correlation between inflammation values (high-sensitivity C-reactive protein) and disease progression (Zhang Y. et al., 2023).

White matter hyperintensities also play an important role in the AD and MCI spectrum. In a multicenter evaluation of automated segmentation algorithms using 3D fluid-attenuated inversion recovery (FLAIR) sequences, deep learning based (re-trained) algorithms performed well (Gaubert et al., 2023).

### 6.2 Frontotemporal dementia

Frontotemporal dementia or frontotemporal lobar degeneration (FTD/FTLD) is a common cause of dementia in patients typically between 45–65 years (Galimberti and Scarpini, 2012). The most frequent phenotype is the behavioral variant frontotemporal dementia (bvFTD) (Rascovsky et al., 2011). Other subtypes are semantic variant PPA (svPPA) and non-fluent variant PPA (nfvPPA). Both sporadic and familial FTD exists. The genetic overlap of bvFTD with amyotrophic lateral sclerosis (ALS) form a special variant called FTD-ALS.

Patients with FTD suffer from different symptoms, e.g., hoarding and obsessive-compulsive behaviors. A related study indicated associations of cortical atrophies of the left temporal lobe, the left insula and the anterior cingulate gyrus with hoarding, while obsessive-compulsive behaviors were associated with cortical decrease in the anterior cingulate, the bilateral hippocampus, and amygdala (Mitchell et al., 2019).

Neuropsychiatric symptoms are most common in FTD and are associated with cortical atrophies in cingulate, insular, and inferior frontal brain areas (Ozzoude et al., 2023). Lesion and/or atrophy of the medial and lateral ventral prefrontal cortex may also increase apathy and other inappropriate behaviors (Huey et al., 2015). Generally, apathy seems to be associated with volume loss of the ventral prefrontal cortex, the posterior cingulate cortex and the adjacent lateral cortex, as well as the superior temporal sulcus in both AD and FTD (Huey et al., 2016).

The association of CSF biomarkers and distinct brain atrophies is not yet sufficiently understood. However, cortical atrophies can be partially explained by levels of Aβ and 14–3-3 in AD, and neurofilament light chain and 14–3-3 in FTD (Falgàs et al., 2020).

The determination of ventricular volume as a simple follow-up parameter in FTD was suggested in a study from Tavares et al. (2019). In particular, the volume of the temporal horns often seems to provide an excellent follow-up parameter for several diseases (Erten-Lyons et al., 2006).

A machine learning approach has shown good differentiation between FTD and other dementias using FreeSurfer segmentation, numerous clinical and MRI data (De Francesco et al., 2023). Differentiation of AD and FTD appears to be possible through the reduced cortical thickness in the posterior cingulate gyrus, which seems to be characteristic of typical and atypical AD, but not FTD (Lehmann et al., 2010). In one study, FTD patients had a more selective loss in frontal cortex and in anterior parts of the temporal lobes compared with AD patients (Möller et al., 2016).

A longitudinal FreeSurfer study of Alzheimer’s disease and behavioral-variant frontotemporal dementia revealed that, at follow-up, patients with AD demonstrate a pronounced cortical volume loss in the inferior parietal and posterior cingulate cortex, while patients with bvFTD show a greater volume loss in the striatum (Landin-Romero et al., 2017).

A (multi-level) hierarchical classification algorithm of AD versus FTD (and bvFTD versus PPA, and nfvPPA versus svPPA) revealed distinct discriminative areas for each comparison using machine learning and demonstrated an overall accuracy of 75.8% (Kim et al., 2019). A study from Barcelona, which tried to distinguish control, AD, and FTD groups using support vector machines, showed an accuracy of 82% in distinguishing the control and FTD groups, and 63% in distinguishing the AD and FTD groups (the accuracy improves to 75% after adding longitudinal data) (Pérez-Millan et al., 2023a; Pérez-Millan et al., 2023b).

White matter hyperintensities and cortical atrophy are associated with a loss of empathy (Ozzoude et al., 2022). Emotional decline in bvFTD could be triggered by an atrophy of the right pregenual anterior cingulate cortex (Sturm et al., 2013). In 2023, a study revealed significant atrophies of the frontotemporal cortex and the bilateral anterior-dorsal thalamus in sporadic bvFTD (Jakabek et al., 2023). Some patients with bvFTD suffer from extrapyramidal symptoms, which could be caused by brainstem atrophy (Heikkinen et al., 2022).

Repeat expansion within C9orf72 is the most common genetic cause of FTD, which especially seems to be associated with gray matter changes (Popuri et al., 2018), a thalamic atrophy (Bonham et al., 2023) and a loss of brain stem white matter (Pérez-Millan et al., 2023a; Pérez-Millan et al., 2023b). Dyslexia susceptibility genes play an important role in frontotemporal dementia as well and are associated with specific local cortical thickness reduction (Paternicó et al., 2016). In svFTD and nfvPPA, different patterns of cortical atrophy are observed (Rohrer et al., 2009). The rate of brain volume loss in FTD varies depending on the mutation, as demonstrated for MAPT and GRN (Whitwell et al., 2011). Pre-symptomatic mutation carriers could be useful for disease monitoring (Borrego-Écija et al., 2021).

Cortical thinning and regional prefrontal cortical atrophy has also been observed in patients with ALS-FTD (Schuster et al., 2014; Ratti et al., 2021).

### 6.3 Dementia with Lewy bodies (DLB)

Although DLB is the second most common dementia of the elderly (>65 years) (Walker et al., 2015), it seems to be one of the least scientifically understood diseases. One review revealed a lack of detailed understanding of its clinical course, neuropathology, genetic factors, and molecular mechanism (Outeiro et al., 2019). MRI is still only a supportive marker in the diagnostic pathway (McKeith et al., 2017, 2020).

Studies have reported focal pronounced atrophies of the substantia innominata (Hanyu et al., 2007) and the insula (Tisserand et al., 2024). A low hippocampal volume is also associated with a risk of DLB in patients with MCI (Kantarci et al., 2016). But DLB shows significantly larger hippocampal volumes than AD and MCI (Mak et al., 2014, 2017). Atrophy of extra-hippocampal structures linked to visual functions were found in patients with DLB as well (Delli Pizzi et al., 2016). DLB subgroups with psychiatric and cognitive onset showed different atrophy patterns (Hansen et al., 2022) of the substantia innominate. The caudate nucleus appears to be relatively unaffected by global atrophy (Khadhraoui et al., 2022), while the brainstem also atrophies at the same rate (Müller et al., 2023). Gray matter atrophy is associated with decrease in dual task gait in DLB (Subotic et al., 2023).

More prospective and longitudinal studies for the evaluation of MRI (especially volumetric analyzes), FDG-PET, biomarkers, and clinical tools are needed (Hansen et al., 2023a,b; Burgio et al., 2024).

### 6.4 Parkinson’s disease dementia (PDD)

To begin with, a distinction must be made between PD, PD-MCI, and PDD. The worse the cognitive state, the more advanced atrophy is to be expected. A meta-analysis of patients with PD revealed a regional atrophy that mainly manifests in the gray matter (He et al., 2020), but with several limitations. Another study (Říha et al., 2022) reported patients with PD show an accelerated volume loss of the hippocampal, which could be a marker for a dementia conversion (Low et al., 2019). Hippocampal subfield analysis revealed significantly smaller volumes in patients with PD-MCI than in patients with PD but without cognitive impairment (Becker et al., 2021). Additionally, a pronounced cortical thinning was found in PD patients with MCI compared with those without (Mak et al., 2015). A study from Singapore revealed pronounced baseline atrophy of the thalamus and progressive atrophies of thalamus, caudate nucleus, presubiculum, and cornu ammonis 1–3 (Foo et al., 2017). Dopamine loss may support the development of cortical atrophies (Sampedro et al., 2019).

In a four-year follow-up study, cortical thinning was correlated with impairment in visuospatial and visuoperceptual performance (Garcia-Diaz et al., 2018b), while another study found a link between poor test performance and a pronounced cortex atrophy of the lateral temporo-parietal regions (Garcia-Diaz et al., 2018a).

An association of white matter hyperintensities with global brain atrophy and cognitive impairment has been reported (Chen et al., 2020). A mild midbrain atrophy was found in 20% of PD patients (Sako et al., 2023). A more pronounced atrophy of the corpus callosum was found in patients with PDD than in PD and PD-MCI (Goldman et al., 2017). Left-sided olfactory amygdala volume reduction is not only associated with hyposmia but with cognitive impairment in patients with PD and can also predict a possible shift to PDD (Ay et al., 2023). The cortical atrophy of PDD is less severe than that in AD or DLB (Colloby et al., 2020). An asymmetric course with an early left-sided atrophy and late right-hemisphere involvement was revealed in a study from the USA (Claassen et al., 2016).

Therefore, MR volumetry can potentially play a role in the early detection of progression from PD to PDD (Trufanov et al., 2013).

### 6.5 Vascular dementia (VD)

White matter lesions can be visually assessed better in T2-or FLAIR-weighted images than in T1-weighted sequences, which are usually required by segmentation algorithms. The classic Fazekas score (Fazekas et al., 1987) is still used today to simplify assessments, but it has long since ceased to be suitable for fine classification and follow-up monitoring. While Fazekas 0 and 1 are usually not considered VD, a score of 2 can describe early VD, while a score of 3 can represent classic VD. However, there is no fine granular classification in the score, which is needed to describe a progressive disease.

The reasons for such lesions are diverse and range from stroke, arterial hypertension (Sierra, 2014), atrial fibrillation, arteriosclerosis (Kim et al., 2014), and carotid stenosis to rarer diseases of large and small vessels (Chojdak-Łukasiewicz et al., 2021) to genetic diseases such as CADASIL (Kalimo et al., 1999) and CARASIL (Müller et al., 2020). Vitamin D insufficiency is also linked with white matter lesions (Annweiler et al., 2015). Subcortical ischemic vascular dementia (SIVD) is a term that describes a disease with the typical subcortical MR lesions in order to separate it from other causes, like large infarctions (Chui, 2007).

Volumetric approaches, which are significantly more suitable, show an association of measured global lesion volumes with this Fazekas Score (Andere et al., 2022). A combination of T1-and T2-weighted sequences is probably the most accurate way to determine such lesion volumes, otherwise adapted normalizations and metrics are recommended (Valdés Hernández et al., 2017).

A high lesion load must be viewed as a possible cause of dementia (or as secondary or mixed dementia), especially in old people (Jellinger and Attems, 2010). Especially, frontal white matter hyperintensities could have a strong impact in cognitive impairment of older adults (Boutzoukas et al., 2021).

Another reason for neuropsychiatric deterioration in addition to the lesion itself can be the induced focal thinning in connected cortical regions (Duering et al., 2012). Furthermore, a high lesion load has been associated with hippocampal atrophy in mild cognitive impairment in a study from Sweden (Eckerström et al., 2011). A study from China reported cognitive deterioration with abnormalities in the brain network between hippocampal subfields and the whole cerebral cortex (Wang et al., 2018). Silent micro infarction may also play a crucial role (Knopman et al., 2015).

Besides FreeSurfer, several other white matter tools exists, e.g., Brain Intensity AbNormality Classification Algorithm (BIANCA, part of FSL) (Griffanti et al., 2016) and UBO Detector (Jiang et al., 2018). However, the right choice of sequence and segmentation algorithm is essential (Hotz et al., 2022).

A study revealed an association between cortical volume and cognitive impairment in patients with white matter lesions using FreeSurfer (Liu et al., 2021). In addition to the information provided by volumetry, MR perfusion (e.g., arterial spin labelling) can also detect brain areas with reduced blood flow in vascular diseases (Gyanwali et al., 2022).

Stroke-dependent severe neurocognitive decline appears in approx. 10% of patients up to 3 months after stroke (Aamodt et al., 2021). In the years after a stroke, a progressive ipsilateral brain volume reduction has also been observed (Salah Khlif et al., 2022).

Hippocampal lesions may explain memory deficits in patients with VD (He et al., 2022). Hippocampal subfield volumetry via FreeSurfer revealed a significant volume reduction of the left hippocampus, left subiculum, presubiculum, and the right CA4/dentate gyrus in patients with vascular lesions and MCI (Li et al., 2016). Another reason fot such memory impairments could be cortical thinning in the precuneus and medial temporal lobe (Chen et al., 2021).

Regarding white matter lesions, deep learning may be a promising solution to specifically classify, monitor, and evaluate these lesions. A study using VUNO Med-DeepBrain (9F, 479, Gangnam-daero, Seocho-gu, Seoul, Korea) and FLAIR images demonstrated the successful classification via the Fazekas scale and could distinguish non-SVID from SVID (Joo et al., 2022). The main architecture of most deep learning solutions is actually still CNN based (Dong and Hayashi, 2024).

### 6.6 Cerebral amyloid angiopathy (CAA)

The accumulation of amyloid β (Aβ) in the vascular walls of intracranial (micro-) vessels defines CAA as a form of VD (Wang et al., 2024). These deposits can lead to (atypical) brain hemorrhages. CAA patients are usually significantly older and a overlaps with other dementias exist. This may be the reason why no significant subcortical atrophy has been observed in some studies (Chen et al., 2023). However, most studies suggest that CAA also leads to cortical thinning (Subotic et al., 2021). A large study demonstrated significant losses of whole cortical volume as well as bilateral hippocampus, amygdala, thalamus, left caudate and right putamen volumes in patients with positive amyloid status (Ten Kate et al., 2018).

In patients with amnestic MCI, the amyloid status can be predicted by hippocampal volume, grey matter volume, or the ratio of hippocampal volume and whole brain volume (Kang et al., 2020).

### 6.7 Progressive supranuclear palsy (PSP)

PSP as a rare atypical parkinsonism with vertical gaze, pseudobulbar palsy, and dementia (Steele, 1964). Volumes of the thalamus, mesencephalon, and caudate nucleus are significantly reduced in PSP (Coughlin and Litvan, 2020). A study revealed an association of gait characteristics in PSP and volumetric changes using FreeSurfer (Chatterjee et al., 2023). The mild pontine atrophy compared to the pronounced mesencephalic volume loss is used as a diagnostic criterion by many indices along with “neuroradiologic signs” on MRI (Slowinski et al., 2008; Hussl et al., 2010; Mittal et al., 2017; Cui et al., 2020; Lupascu et al., 2023). Additionally, volume loss has been observed in the frontal lobe, particularly the superior frontal gyrus (Worker et al., 2014).

Despite a detailed fine segmentation of the brain stem, deep learning methods could improve the early detection of patients (Nigro et al., 2024).

### 6.8 Multiple system atrophy (MSA)

MSA is a rare synucleinopathy, characterized by α-synuclein-positive cytoplasmic deposits. It presents with Parkinsonism and is challenging to diagnose for both neurologists and neuroradiologist (Goh et al., 2023). It is separated into Parkinsonian (MSA-P) and cerebellar (MSA-C) subtypes; atrophies of the putamen, middle cerebellar peduncles, pons, and cerebellum are described. However, a study did not detect significant volume reductions in cortical morphology for MSA compared with that for PD and control groups (Worker et al., 2014).

In addition to detailed brain stem segmentation, a deep learning approach also shows promise for the future detection of this disease.

### 6.9 Alcohol dementia/alcohol use disorder

A common secondary disease that also leads to cortical atrophy and can be a disruptive factor is alcohol addiction. The expected volume losses are in the left ventral diencephalon, left inferior and middle temporal gyrus, left caudate nucleus, brain stem, and cerebellum (Squeglia et al., 2014). Interestingly, one study here even describes a possible regional recovery of brain volume during abstinence (Durazzo et al., 2023). Additionally, a thickness reduction of the occipitotemporal cortex and an association with apathy was reported (Yang et al., 2020). Hippocampal atrophies, particularly of the subiculum, CA1, molecular layer, and hippocampal tail, have also been observed (Sawyer et al., 2020).

### 6.10 Other dementias

Other forms of dementia are very rare and only poorly investigated using MR morphometric methods. An exception is diseases with a specific atrophy pattern. These include also mixed etiologies, for example, semantic-variant primary progressive aphasia (svPPA) and posterior cortical atrophy (PCA) (Fazlollahi et al., 2023), which are subtypes of FTD, corticobasal degeneration and AD, respectively.

Even diseases that are not primarily referred to as dementia can present this as a secondary consequence. The most prominent example is multiple sclerosis. Besides the thalamic changes, the cortical thickness is significantly reduced in older patients with multiple sclerosis and cognitive impairment (Jakimovski et al., 2023). An association between whole brain volume and disability exists as well (Moridi et al., 2022).

A study revealed a cortical involvement in idiopathic normal-pressure hydrocephalus (Bianco et al., 2022). This form of dementia is also suitable for segmentation algorithms. In addition to calculating the volume of the ventricles, a measurement of the areas “compressed” by the increase in CSF is also of interest but remains underresearched.

## 7 Discussion

There are similar reviews about brain segmentation (Singh and Singh, 2021) or hippocampal segmentation software (Zhang J. et al., 2023). Our review provides an up-to-date status of the software and dementias researched so far with a focus on FreeSurfer. It makes it possible to discover numerous gaps in research and to focus specifically on a question that has not yet been researched.

Even if numerous commercial and non-commercial software solutions for automated brain segmentation and volumetry exist, FreeSurfer seems to be currently the most frequently used. There are many reasons for this. In addition to the extensive functions for almost all questions and diagnosis, regular updates are also offered. The accuracy of the tool is sufficient. Since it has been around for a long time, there is a wide acceptance and validation. In addition, it is free and there is a large open source community that is constantly adapting the extensive documentation. FreeSurfer is compatible with many other tools (e.g., FastSurfer, CerebNet).

There are still numerous gaps in research. Be it the few publications in the area of Lewy Body Dementia, which has only been sparsely researched, or the multiple atrophy patterns in Alzheimer’s disease, which are still not fully understood. Many diseases are underrepresented, measured by the percentage ratio of entries found compared to the prevalence of the disease. There are also only a few longitudinal studies that have been conducted using the same protocols and MRI devices. The many new artifacts in clinical application in 7 T MRI will also influence the segmentation algorithms.

Increasing comorbidities and mixed dementias in old age, as well as the normal level of physiological brain involution, are areas of research that will occupy us for decades to come.

In addition to volumetry and nuclear-medicinal examinations, there are also new possibilities for quantification in MRI using T1-and T2-mappings (Gräfe et al., 2022; Müller et al., 2022) or quantitative susceptibility mapping (Li et al., 2024). Improvement from 3 to 7-Tesla scanning also promises more accurate diagnostics.

### 7.1 AI-based software/algorithms

Many of the methods mentioned, such as FreeSurfer, are based on neural networks and are formally already AI software. Nevertheless, other AI algorithms can additionally be applied to all the methods mentioned, potentially facilitating new discoveries in the field. In particular, when networking multiple different data, such as clinical information (Noroozi et al., 2024), electroencephalogram (Carrarini et al., 2024), CSF biomarkers and MR imaging data, enormous advantages can arise from AI approaches. Of course, as the number of data to be processed increases, so does the computing power and time required.

However, a major problem remains the diversity of data, MR sequences, and scanners, which make uniform, large, multi-center data analysis difficult. Here, too, the advantage of neural networks could become apparent, as they already include a very efficient normalization of the data.

Besides the brain, the liver is another organ where segmentation using AI can deliver promising results (Zhang et al., 2024), e.g., universal models like segment anything model (SAM), MedSAM and SAMed2D in hepatocellular carcinoma (Saha and Van Der Pol, 2024).

### 7.2 Limitations

Today, neurodegenerative disorders that progress to dementia are often identified solely from a clinical perspective (Tahami Monfared et al., 2023), without considering the underlying biological substrate, such as the CSF biomarker profile. This is an important (disturbing) factor that can also lead to incorrect diagnoses and inclusions or exclusions within many studies. In the case of small deviations in median brain volumes for some diseases, such misclassifications could also influence the validity of studies.

Due to the heterogeneity of the diseases and the software tools used, it seems almost impossible to conduct a homogeneous PubMed search in this research area. Many programs are only used for individual diseases and are specifically adapted for them, while a universal solution for whole brain volumetry with specialization in certain regions using additional scripts/apps, such as those offered by FreeSurfer, has not yet been fully adopted by the research community.

We therefore concentrated on the FreeSurfer results. Accordingly, a certain bias in the searches with an emphasis on the results in favor of FreeSurfer and Alzheimer’s disease is to be expected.

To reduce the potential of underrepresentation of certain dementia types and overlooking relevant software tools, we performed a deep search on all software tools found. Nevertheless, there remains a certain residual risk of having overlooked or underestimated software solutions.

In addition, we did not perform a detailed evaluation of accuracy and reliability, as the latter in particular was often not available and the sensitivity/specificity data often referred to specific comparisons of two patient cohorts, which were, however, often defined differently in the studies. This significant variability in study protocols affects result comparability, and therefore, a detailed evaluation of the accuracy and reliability of segmentation tools is almost impossible.

## 8 Conclusion

Automated brain segmentation and volumetry could enable earlier and more reliable dementia diagnosis than other approaches. It can also clarify and objectify the radiological findings. However, the method is not yet widely established. There is also a lack of studies proving its high diagnostic accuracy. In everyday clinical practice, MR volumetry still plays little role in smaller hospitals and is mainly carried out by university institutions for research and validation purposes. The importance of automated evaluation in diagnostics will continue to increase in the coming years. Nevertheless, the clinical picture, CSF biomarkers and PET will remain important.
