# Supplementary figures and images for: Automated brain segmentation and volumetry in dementia diagnostics: a narrative review with emphasis on FreeSurfer
Source: Front Aging Neurosci. 2024 Sep 3;16:1459652. doi: 10.3389/fnagi.2024.1459652 (PMC11405240; doi:10.3389/fnagi.2024.1459652)

**
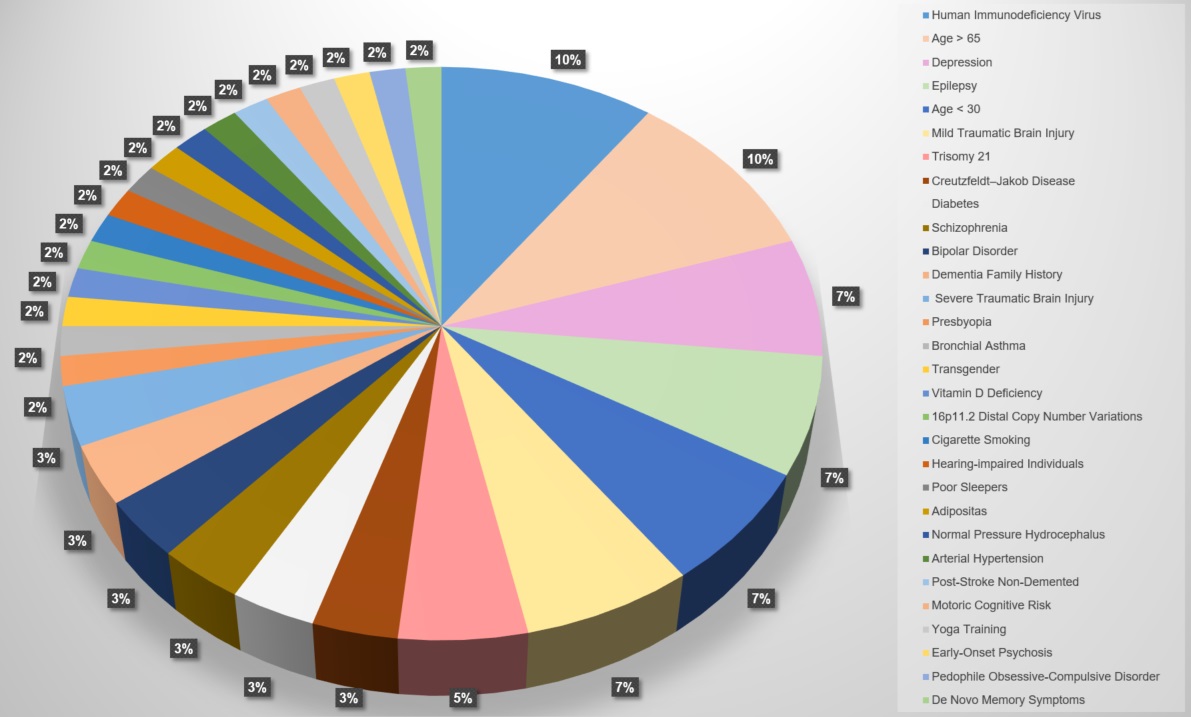
**

**Supplemental Figure 1.** Pie chart showing the population of the “Other” cohort in Fig. 1.

Supplement: Supplementary file 1 [file Table_1.DOCX]
